# Exploring the Intensity, Frequency, and Duration of Pediatric Constraint Induced Movement Therapy Published Research: A Content Analysis

**DOI:** 10.3390/children9050700

**Published:** 2022-05-10

**Authors:** Bryan M. Gee, Sharon Leonard, Kimberly G. Lloyd, L. Derek Gerber, Hannah Quick, Taylor Raschke, Justin Yardley, Jacob D. Earl

**Affiliations:** 1 Department of Occupational Therapy, Rocky Mountain University of Health Professions, Provo, UT 84606, USA; sharon.leonard@rm.edu (S.L.); kimberly.lloyd@rm.edu (K.G.L.); 2 Department of Physical Therapy, Idaho State University, Pocatello, ID 83021, USA; derekgerber@isu.edu; 3 Department of Occupational Therapy, Idaho Falls Community Hospital, Idaho Falls, ID 83404, USA; hmquickotr@gmail.com; 4 TheraSens Inc., Monterey, CA 93940, USA; raschketaylor1@gmail.com; 5 Department of Occupational Therapy, Logan Regional Hospital, Logan, UT 83841, USA; justin.yardley@imail.org; 6 Dixie Regional Medical Center, St. George, UT 84970, USA; jacob.earl@imail.org

**Keywords:** pediatric, constraint induced movement therapy, dosage, motor learning

## Abstract

Constraint Induced Movement Therapy (CIMT) utilizes a behavioral approach to neurorehabilitation involving constraint of an unaffected upper extremity which forces the use of the affected extremity. There is substantial evidence supporting the effectiveness of CIMT among both children and adults. The purpose of this study was to explore the frequency, intensity, and duration parameters across the published clinical outcomes related to pediatric CIMT (pCIMT) among children and youth populations. A content analysis approach was used to search the following databases Google Scholar, OT seeker, American Occupational Therapy Association special interest section, Medline, EbscoHost, and Cinhal. A total of 141 studies were identified via the initial search, with 51 studies meeting inclusion criteria. The findings revealed that 100% of the studies included restraint of the non-affected upper extremity, 73% incorporated repetitive task-oriented training, but less than half prescribed home practice strategies. Further, only 34% of the studies reviewed included all three components of CIMT. Outpatient hospital clinics and home-based settings were the most utilized settings for research studies. The mean minutes per session was *M* = 205.53, *SD* = 164.99. As part of the plan of care, the duration and frequency of therapy both had similar means (~*M* = 3.60) and standard deviations (~*SD* = 1.65). There was a significant variance of hours during (*SD* = 139.54) and outside of therapy (*SD* = 130.06). The results of this study, together with other emerging evidence, can assist practitioners in prescribing dosages dependent on the setting, the pediatric client, and their current functional status.

## 1. Introduction

Constraint-induced movement therapy (CIMT) has been ubiquitous in the physical medicine practitioner’s repertoire since its inception in the 1990s. Taub et al. [1] developed CIMT for neurologically motor impaired adults to combat learned non-use of an affected upper extremity. Essentially, CIMT utilizes a behavioral approach to neurorehabilitation involving constraint of an unaffected upper extremity which forces the use of the affected extremity. Taub and colleagues hypothesized that the improvements from CIMT were a result of cortical reorganization and axonal sprouting [1].

In the late 1990s pediatric CIMT (pCIMT) was introduced into neurorehabilitation by multiple teams of researchers and therapists [2]. Hoare et al. [3] determined the following; constraint of the unaffected upper extremity (UE); dosing, shaping and repetition; and the child’s natural environment as essential elements for successful implementation of a pCIMT program. Individuals who experience neurological damage that results in hemiparesis or hemiplegia have been observed to display learned nonuse and developmental disregard, both of which reduce spontaneous use and function of the affected UE [4]. The observed phenomenon that has been characterized as learned nonuse is when a client has “repeated unsuccessful efforts with voluntary movement post injury [4] (p. 23). Developmental disregard is a phenomenon where a pediatric client neglects to attend to the affected UE and may perceive the UE as not being present” [4] (p. 23). Both phenomena are primary targets of CIMT, which pCIMT aims to reduce [4].

Rehabilitation specialists (occupational and physical therapists) have used various types of constraints for the unaffected upper extremity, including mitts, gloves, slings, splints, and casts. In addition, treatment settings for individualized and group therapy sessions have included the home, outpatient clinics, and specialized intensive therapy camps [4].

### 1.1. Types and Parameters of CIMT and pCIMT

As CIMT and pCIMT continued to evolve over time with varying model types, a uniform terminology was necessary to clarify categories for knowledge translation, recommendations, and for future research.

- Signature CIMT (sCIMT) is Taub’s original model developed for adults with hemiparesis following stroke. This involves restraint of the well-functioning upper limb for 90% of waking hours for at least two weeks and the addition of intensive training of the involved upper limb for several hours per day [5,6];
- Modified constraint-induced movement therapy (mCIMT), like the signature model, contains restraint and intensive training. However, mCIMT varies parameters of restraint used for the well-functioning upper limb (sling, cast, mitt/glove); the type of structured training (shaping/repetition, motor learning); the program duration (hours per day) and length (number of weeks); and the location, context, and provider of training (home/camp, individual/group, therapist/parent) [5,6];
- Forced use therapy involves restraint of the well-functioning upper limb but no specific structured training is provided. Forced use is related to, but not considered CIMT [5,6];
- Ramey et al. [4] proposed that pCIMT includes four conditions: constraint of the less or unimpaired UE; use of specific techniques to help shape, refine and practice functional movements of the highly intensive systematic training; delivered in a naturalistic setting; and a plan to support the transfer of learning and generalization of skills to activities of daily living [4];
- Modified pCIMT has been differentiated from traditional pCIMT only in the dosage by reducing the number of systematic trainings per day (intensity) and modifying the number of consecutive days of the restraint applied to the non or less impaired UE [4].

Eliasson et al. [5] further pointed out that, regardless of which model of CIMT is used, two criteria must be satisfied to be considered CIMT: (1) restraint of the functioning upper extremity and (2) an established intensive and structured program.

Duration of pCIMT varies. As a part of their systematic review, Hoare et al. [3] reported calculated dosage times of pCIMT as follows: the total number of hours combining the therapist-led hours, parent-led hours, other hours, and forced use. The authors discovered the mean number of hours provided across all the studies reviewed was 129 h, ranging from 20 to 504 h. When the forced use component was removed, the average total dosage across included studies was 79 h (ranging from six to 210 h). The average length of a CIMT program provided was over five weeks, ranging from one week to a maximum of 12 weeks. The length of daily sessions across studies ranged from 0.5 to eight hours per day. The frequency of therapist and or parent led sessions ranged from two to seven days per week. Lastly, an average of 56 h of pCIMT was led by a therapist (0–126 h) [3].

### 1.2. Efficacy of pCIMT

A plethora of existing research demonstrates pCIMT as an effective and safe intervention for children and youth. A thorough systematic review of 36 studies conducted by Hoare et al. [3] included 1264 children with unilateral cerebral palsy (CP). Measured outcomes were classified into primary and secondary outcomes to illustrate the effects of pCIMT. The primary outcomes focused on valid and reliable measures for bimanual, unimanual, and manual functioning. Secondary outcomes, which are gains resulting from the sequela of the intervention, comprised individualized measures of performance, self-care, body function, participation, quality of life, and parenting and family measures. The authors concluded the mode of pCIMT is an ancillary matter compared to the importance of ensuring an intensive, target specific, and fully supported program.

Content analysis of literature has become a recent trend in the rehabilitation sciences and medicine [7]. Analyses have focused on pediatric outcomes studies in occupational therapy [8], constraint induced movement therapy among adults [9], qualitative research on children and youth with Autism [10], and personal factors of the International Classification of Functioning, Disability, and Health [11].

Exploring more recent pCIMT scholarship, Deluca et al. [12] demonstrated clinically significant new functional skill development after an initial pCIMT treatment in 28 children with CP and a mean age of 31 months. The reported pCIMT treatment included 3 h per day, many days per week, up to four weeks. In addition, this study revealed children continued to produce additional substantive functional gains after undergoing multiple sessions of pCIMT. More recently, Ramey et al. [13] reported clinically significant gains among 118 children between the ages of 2 and 7 years. The authors implemented a high-dose pCIMT intervention (3 h per day, 5 days per week, for 4 weeks), and produced a pattern of greatest short- and long-term improvement and significant gains in various functional outcome measures [12]. The research exploring the efficacy for pCIMT continues to demonstrate clinical significance using more rigorous research designs and larger samples, which supports, in part, the primary aim of this study and will be further compared to the findings from this study.

The primary aim of this study was to explore common frequency, intensity, and duration parameters of published clinical outcomes related to pCIMT among children and youth populations. A secondary aim of this study was to inform rehabilitation practitioners of settings where pCIMT is occurring to guide future planning for program development in diverse practice settings. This study was conducted between 2017 and 2019, compliments the more recently published literature mentioned above (that was not available during data collection), and adds evidence of appropriate forms and parameters of pCIMT.

## 2. Materials and Methods

### 2.1. Procedures

This study was not deemed as research that involved human subjects and was approved by the Human Subjects Committee at Rocky Mountain University of Health Professions protocol #2022-38. As a part of the study methodology, authors operationalized key terms as part of the study. Pediatric Constraint Induced Movement Therapy (pCIMT) was defined as an intervention for children (from age one up to age 21) in which the unaffected upper extremity was restricted, and the affected upper extremity was principally used during therapeutic activities. The foundational components of pCIMT included repetitive, task-oriented training (consisting of shaping or repetitive task practice), home practice strategies (transferring gains made in therapy into real experiences and activities at home), and constraint of the non-affected limb (restraint of the less impaired UE to encourage the use of the more affected UE during tasks) [1,4,14]. An outcome study was defined as any study that takes patient experiences, preferences, and values into account and is intended to provide scientific evidence relating to decisions made by all who participate in health care [15].

Intervention frequency was defined as the number of treatment sessions undergone by the patient that included pCIMT as the sole intervention or one of a few interventions as part of a given session [11]. Intervention intensity referred to the length of each intervention session reported in minutes [16,17,18,19]. Lastly, the intervention duration was the length of the intervention plan reported in weeks or days [19,20,21,22].

### 2.2. Inclusion Criteria

For an article to be included in the study, it had to meet the following inclusion criteria: (1) the study population had to consist of children that were one to 21 years of age; (2) studies published outside of the years between 2002 and 2019 were omitted; (3) the study design had to be quantitative empirical studies consisting of randomized control trials, quasi-experimental, pre-posttest measures, and report positive intervention outcomes (statistically significant (*p* = 0.05)); (4) the interventions in the study could consist of any of the three main components of CIMT, but required the third component of the constraint of the less impaired limb to be present; and (5) the study had to report at least one of the following aspects related to the intervention being tested, the number of treatment sessions, session frequency per week, duration of the treatment plan, and/or the total hours spent on the intervention. Systematic reviews and meta syntheses studies were excluded as they had insufficient information related to parameters of dosage in method sections of the individual studies included.

### 2.3. Searching

**Figure 1:**
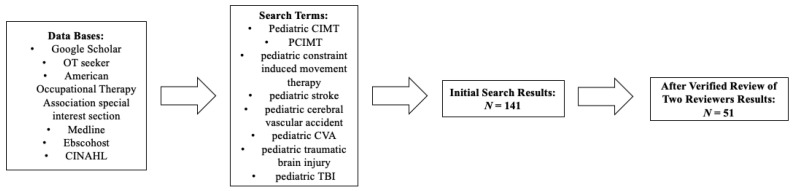
Search and Review Sequence.

The research team consisted of three faculty members and four graduate research participants. Members of the research team were assigned scholarly databases to search using the following terms: Pediatric CIMT, pCIMT or pediatric constraint-induced movement therapy, pediatric stroke, pediatric cerebral vascular accident, pediatric CVA, pediatric traumatic brain injury, and pediatric TBI. The databases searched included Google Scholar, OT seeker, American Occupational Therapy Association special interest section, Medline, EbscoHost, and Cinhal. A total of 141 studies were identified via the initial search, with 51 studies meeting inclusion criteria (refer to Figure 1).

### 2.4. Method of Analysis

Using a spreadsheet program, frequency distributions and descriptive statistics (mean and standard deviation) data of the three components of dosage (intensity, frequency, and duration) across the 51 outcomes studies were analyzed (see Table 1, Table 2, Table 3, Table 4 and Table 5). Data were further categorized according to the treatment setting (outpatient hospital clinic, inpatient hospital, school-based, home-based, day camp, and a university lab).

## 3. Results

Fifty-one peer reviewed outcome studies were included in the study, which yielded a mean number of participants across the studies *M* = 23.92 (refer to Table 1). In assessing the fidelity of pCIMT [11], 100% of the studies included restraint of the non-affected upper extremity, 73% incorporated repetitive task-oriented training, but less than half prescribed home practice strategies. Further, only 34% of the studies reviewed included all three components of CIMT (refer to Table 2).

Evaluation of frequency, intensity, and duration of pCIMT produced notable findings. The mean minutes per session (a therapy session during which pCIMT was utilized) was *M* = 205.53, *SD* = 164.99. The large amount of variance in this aspect of dosage was likely due to pCIMT being implemented at home and at summer camps on the high end and outpatient hospital clinics on the low end. The duration and frequency of therapy, as a part of the plan of care, both had similar means and standard deviations. The significant variance of hours during (*SD* = 139.54) and outside of therapy (*SD* = 130.06) is notable (refer to Table 3).

The distribution of settings where pCIMT occurred revealed outpatient hospital clinics and home-based settings were the most utilized settings for research studies. This is not surprising as applying and wearing a restraint in these settings would likely have been most confident and controlled (e.g., therapist or caregiver present) for the researchers (refer to Table 4).

Most of the studies reviewed used designs that can be categorized as level II [23], specifically randomized control trials (41%). However, the overall distribution of studies included less rigorous design levels III and IV (59%) (refer to Table 5).

## 4. Discussion

The primary aim of this study was to explore common frequency, intensity, and duration parameters of published clinical outcomes related to pCIMT among children and youth populations. A secondary aim of this study was to inform rehabilitation practitioners regarding settings where pCIMT is occurring to guide planning for future program development in diverse practice settings.

This study’s results differed from those of Hoare et al. [3] in several parameters. Hoare et al. reported mean number of hours of pCIMT across studies as 129 h which included the total number of hours from therapist-led, parent-led, other hours, and the time spent implementing forced use. Conversely, our investigation found mean number of total hours of skilled therapy was 63 h. Hoare et al. [3], reported the average length of a CIMT program provided was five weeks where our findings showed an average length of 3.5 weeks. Hoare et al. 2019, reported the average daily intervention across all studies was found to be 0.5 to eight hours per day where our study reported the average length per session was approximately 200 min or 3.3 h. However, Hoare et al. [3], found the interventions were implemented between two to seven days per week and our study reported an average of 5 days per week. Lastly, an average of 56 h of the CIMT program led by a therapist (0–126 h) was reported by Hoare et al. [3]. Our study found a notable difference with an average of 130 h of therapist led intervention which is on the high end of the range of their study.

Furthermore, comparing the dosage findings from this study to those of Deluca et al. [12] and Ramey et al. [7], recent randomized control trials implemented pCIMT dosages at 3 h per day, 5 days per week and approximately 20 days [12] and 28 days [13]. Two findings support their dosage parameters, specifically, the days per week (*M* = 4.94) and the number of weeks per plan of care (*M* = 3.61). This alignment is helpful for clinicians implementing the intervention in non-research settings or focus.

Much like a systematic review, the findings from this content analysis provide a comprehensive resource for clinicians regarding the status of pCIMT dosage, which may be useful for some clinicians who lack resources (time, access to databases, or full-text studies) focused on pCIMT.

Moreover, this study provided additional information related to the intervention setting, which is helpful to rehabilitation professionals in deciding if pCIMT may be an intervention that could be successfully delivered in their own setting. The findings provide clinicians with crucial points of dialog and clinical reasoning when establishing dosage within their practices or in novel settings, e.g., school-based contexts, early intervention/homes, and emerging practice settings, like summer day camps, where dosage may be further controlled.

In addition, this study provides researchers with information related to the fidelity of CIMT intervention. A method to enhance intervention of pCIMT within an experimental group would be to ensure implementation of home practice strategies by parents and caregivers.

### 4.1. Limitations

This content analysis of dosage parameters within pCIMT contained the following limitations. The study was conducted between 2017 and 2019 and newer literature emerged on the topic of dosage and pCIMT [3] that was not included as a part of the conceptualization and design of the study. The study did not include the range of significant outcomes into categories of effectiveness and/or effect size. More importantly, the study did not track the type and frequency of the outcome measures used in each study. This type of data would have helped practitioners explore different functional outcome measures as they implement their pCIMT programs in their clinics/settings. Additionally, tracking the manual ability MACS and/or gross motor functional classification system levels of the participants (if reported). This additional information would have helped develop a client profile for pCIMT.

### 4.2. Future Research

The purpose of this content analysis was to identify and explore the dosage parameters of pCIMT. The results brought up as many questions as answers and opened the doorway for further analysis of dosage outcomes. For increased value of future research, skilled therapists who are practicing pCIMT need to be documenting and participating in research to create a standardized evidence-based protocol for pCIMT implementation. Comparison of outcomes from research implementing traditional pCIMT and modified pCIMT may help therapists better understand the role of the components of pCIMT. Further research that documents the outcome measures used and each participant’s manual ability and gross motor function to develop a patient profile that may be more responsive to the intensive nature of pCIMT is warranted.

## 5. Conclusions

Rehabilitation specialists and providers who implement pCIMT into practice need to be aware of effective parameters of pCIMT. Currently, there is no standardized protocol for implementing pCIMT; therefore, providers may fall into habitual, and possibly ineffective, routines when providing pCIMT. The results of this study, together with other emerging evidence, can assist practitioners in prescribing dosages dependent on the setting, the pediatric client, and their current functional status. However, rehabilitation specialists should be thinking more about dosage and what constitutes an efficacious and ultimately an effective dosage in pCIMT; thus, more research is warranted.

## Figures and Tables

**Table 1:** Study Sample Size.

Sample	Mean	Standard Deviation
*N* = 51	*M* = 23.92	*SD* = 20.04

**Table 2:** Fidelity to the CIMT Protocol.

CIMT Protocol Fidelity (Morris, Taub, & Mark, 2006) [14]		
Repetitive Task Oriented Training	34/46	73%
Home Practice Strategies	20/46	43%
Restraint	46/46	100%
Total	16/46	34%

**Table 3:** Frequency, Intensity, Duration of Intervention.

	Mean	Standard Deviation
Minutes Per Session	*M* = 205.53	*SD* = 164.99
Duration in Weeks (for plan of care)	*M* = 3.61	*SD* = 2.39
Frequency per week	*M* = 4.94	*SD* = 1.65
Hours of Constraint (during therapy)	*M* = 130.75	*SD* = 139.54
Total Hours of Constraint Outside of Therapy (per plan of care)	*M* = 67.54	*SD* = 130.06
Total Hours of Skilled Therapy	*M* = 63.93	*SD* = 76.64

**Table 4:** Intervention Setting.

Intervention Setting	
Outpatient Hospital Clinic	22
Inpatient Hospital	2
School-Based	2
Home-Based	11
Day Camp	8
University-Lab	1

**Table 5:** Level of Evidence.

Level of Evidence (Portney, 2020) [23]	
Level I	0
Level II	21
Level III	17
Level IV	13
Level V	0

## Data Availability

All data are found within the article.
